# Inducible displacement of cementless total knee arthroplasty components with conventional and weight‐bearing CT‐based radiostereometric analysis

**DOI:** 10.1002/jor.26017

**Published:** 2024-11-15

**Authors:** Rebecca A. Hext, Bart L. Kaptein, James L. Howard, Brent A. Lanting, Matthew G. Teeter

**Affiliations:** ^1^ Department of Medical Biophysics Schulich School of Medicine & Dentistry, Western University London Ontario Canada; ^2^ Department of Orthopaedics Leiden University Medical Center Leiden Netherlands; ^3^ Division of Orthopaedic Surgery Schulich School of Medicine & Dentistry, Western University London Ontario Canada

**Keywords:** implant fixation, inducible displacement, weight‐bearing computed tomography

## Abstract

Aseptic loosening remains one of the top causes of revision surgery of total knee arthroplasty (TKA). Radiostereometric analysis (RSA) is used in research to measure implant migration, however limitations prevent its clinical use. New methods have allowed the same measurements as RSA to be performed with computed tomography (CT) scanners (CT‐RSA). The objective of this study is to determine inducible displacement measurements from weight‐bearing computed tomography (WBCT) and conventional RSA to assess implant stability. Participants (n = 17) completed RSA exams in the supine and standing position, and WBCT exams in the seated (leg extended) and standing position. Double examinations were performed in the seated (WBCT) or supine (RSA) positions. Inducible displacements were measured with model‐based RSA (MBRSA) for RSA exams, and a novel CT‐RSA software, V3MA, for WBCT exams. Precision of each technique was calculated between double examinations. Precision data for tibial component total translations and rotations were 0.05 mm and 0.118°, respectively with WBCT‐RSA, and were 0.108 mm and 0.269°, respectively with MBRSA. MTPM precision was 0.141 mm with WBCT‐RSA and was 0.168 mm with MBRSA. Inducible displacement MTPM of the tibial component was 0.244 ± 0.220 mm with WBCT‐RSA and 0.662 ± 0.257 mm with MBRSA. Inducible displacement measurements with MBRSA were significantly different from WBCT‐RSA for tibial component anterior tilt (*p* = 0.0002). WBCT‐RSA demonstrated comparable precision to MBRSA, and both techniques measured inducible displacements consistent with stable components. Clinical Significance: As the availability of WBCT increases, its use as an alternative to MBRSA is supported to measure the instantaneous fixation of implant components.

## 1 INTRODUCTION

Primary total knee arthroplasty (TKA) procedures are projected to increase more than 450% by 2060 in the United States, leading to the increased burden of revision TKA.^1^, ^2^ Revision surgeries are a major decision, yet this decision is further complicated if the diagnosis is for suspected loosening. Radiographs are used for diagnosis, but they cannot assess for early indications of loosening, and require proper alignment to visualize radiolucent lines indicative of loosening.^3^, ^4^, ^5^ Radiostereometric analysis (RSA) is used to assess implant migration as an indicator of fixation, although its limitations concentrate its use to research participants.^6^, ^7^, ^8^, ^9^ RSA requires the intraoperative insertion of bone markers, uses facilities with specialized equipment and trained personnel, and data may still be lost if the bone markers are occluded by the metal implant.^7^, ^10^, ^11^, ^12^ These limitations motivate the use of a more accessible clinical method to accurately assess aseptic loosening.

New methods have been introduced that allow migration measurements to be performed with clinical computed tomography (CT) scanners (CT‐RSA).^13^, ^14^, ^15^, ^16^ CT‐RSA relies on CT scanners that are widely available and does not require bone markers, mitigating the limitations of RSA. Inducible displacement is the magnitude of micromotion that occurs between loaded and unloaded scans at a single timepoint and can be measured with RSA.^17^, ^18^, ^19^ It is thought to relate to how well‐fixed a component is and can be used to assess the instantaneous fixation of implant components.^19^, ^20^, ^21^ Over the past decade, the availability of weight‐bearing CT (WBCT) has been increasing and provides the unique opportunity to study joints under loading to better understand joint biomechanics.^22^, ^23^, ^24^, ^25^ There is now the chance to combine CT‐RSA and WBCT to produce “WBCT‐RSA” to assess instantaneous component fixation with inducible displacement exams.

Prior studies have shown comparable precision between RSA and CT‐RSA for total hip arthroplasty components or phantom knee studies.^7^, ^10^, ^11^, ^14^, ^26^, ^27^ However, none to our knowledge have clinically assessed inducible displacement for TKA components using WBCT‐RSA and conventional RSA. Therefore, the objective of this study was to determine inducible displacement measurements of cementless TKA components at 5 years postoperation using WBCT‐RSA and conventional RSA.

## 2 METHODS

**Figure 1:**
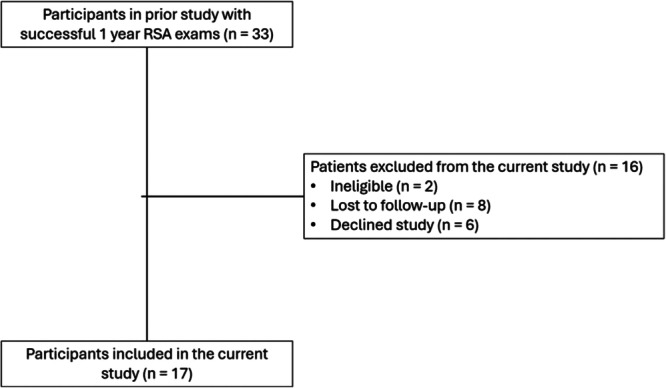
Flow diagram for recruitment to the current study. Participants from the prior study were excluded if they had occluded markers or missed the 1 year follow‐up. All 33 participants with successful 1 year follow‐up RSA exams were invited to the current study.

Participants (*n* = 17) were recruited from a previous RSA cohort that examined a gap balancing versus measured resection surgical technique through 1‐year postoperation (Figure 1). The inclusion criteria and results for this cohort have been published.^28^ All participants received an identical fixed bearing, cruciate‐retaining peri‐apatite coated cementless femoral component and a pegged highly porous cementless tibial baseplate with a condylar stabilizing tibial insert (Triathlon Tritanium, Stryker, Mahwah, NJ). Up to 15 tantalum beads were placed in the proximal tibia and up to 12 were placed in the distal femur intraoperatively to enable RSA evaluation. Postoperative protocols were identical for all participants.

For the current prospective cohort study, participants were invited to return at 5 years postoperation (levels of evidence–Level II). Inclusion criteria was successful RSA images at 1‐year postoperation to enable longitudinal migration measurements and exclusions were the inability to complete questionnaires or imaging examinations. Ethics approval was obtained from our institutional ethics review board and all willing participants provided written informed consent before participation (ClinicalTrials. gov ID#: NCT05877261). The 17 included participants had a mean age at 5 years postoperation of 66.5 years (range, 54–78 years) and a mean body mass index of 36.0 (range, 24.9–49.7).

A conventional supine RSA exam was acquired at 2 weeks, 6 weeks, 3 months, 6 months, 1‐year, and 5 years postoperation using a uniplanar calibration cage (RSA Biomedical, Umea, Sweden). At 5 years postoperation, a second supine exam was acquired shortly after the first for double examination (precision) measurements, as well as a standing exam on both legs with the majority of the participants' body weight going through the operative limb. This was determined by the participant leaning over the operative limb and only using the contralateral limb for balance. The RSA coordinate system was defined with positive translation as proximal translation in the y‐axis, medial translation in the x‐axis, and anterior translation in the z‐axis. Positive rotations were defined as internal rotation about the y‐axis, anterior tilt about the x‐axis, and valgus rotation about the z‐axis. Measurements were adjusted for a right‐sided implant.

Model‐based RSA (MBRSA v4.2, RSAcore, Leiden, The Netherlands) was used to measure movement between supine exams as maximum total point motion (MTPM), defined as the largest movement of a point on the component of interest relative to the bone.^29^ MBRSA was also used to measure movement between the 5 year double supine examinations, as precision, and between the first supine exam at 5 years postoperation and the standing exam at this timepoint, as inducible displacement. Precision was reported as 1.96 times the standard deviation of these values. Measurements were reported as MTPM, and translations and rotations in the X (medial‐lateral), Y (superior‐inferior), or Z (anterior‐posterior) axis. Total translation and total rotation, calculated using the Pythagorean theorem, were also reported. Total translation summarizes the translation vector of the implants center point, however MTPM measures the translation vector for the point on the component with the greatest movement.^30^ The average condition number was 34.6 (range, 15.8–80.2) for tibial bead alignment, and 58.8 (range, 26.0–139.2) for femoral bead alignment. One participant for the tibial component and four for the femoral component had condition numbers above 150 for longitudinal migration exams and were excluded for the respective analyses.^30^ The upper limit of 0.35 mm was used for the rigid body error, and mean rigid body error was 0.11 mm, indicating the tantalum bone markers are stable between exams.^30^

**Figure 2:**
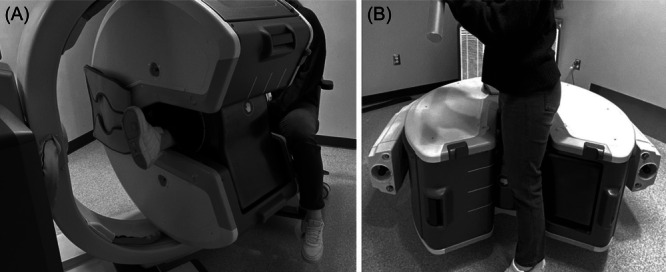
WBCT set‐up for the seated (leg extended) exam (A) and the weight bearing standing exam with the majority of the patient's weight going through the operative limb (B). The WBCT bore is rotated between exams.

At 5 years postoperation, two seated WBCT exams (Figure 2A) and one standing exam (Figure 2B) were acquired (OnSight 3D Extremity CT System, Carestream, New York, United States). A defined WBCT protocol was applied with the following parameters: 90 kVp, exposure 20–41 mAs, slice thickness 0.26 mm, increments 0.26 mm, rotation time 10 s, approximate dose 0.04–0.06 mSv, pixel spacing 0.26/0.26, 884 slices, and a matrix of 1024 × 1024.

**Figure 3:**
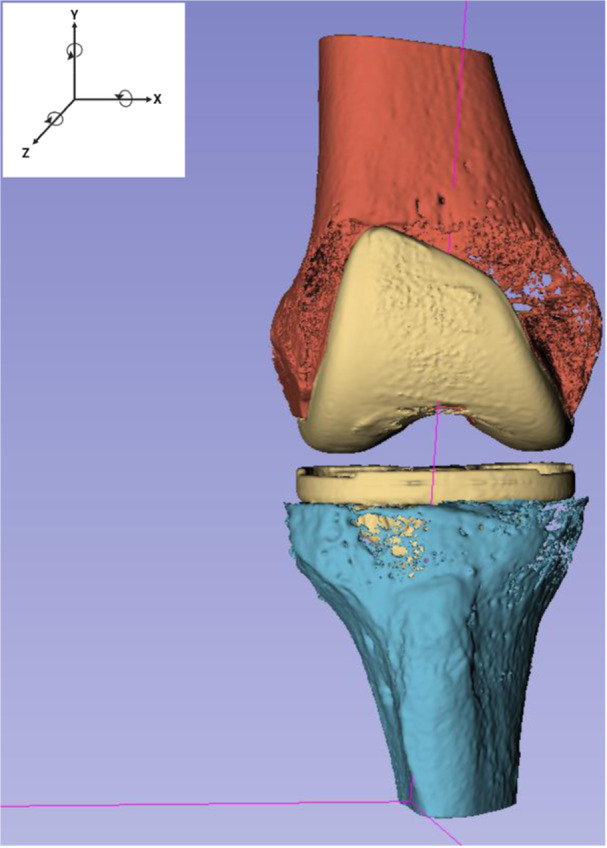
Segmentations of the tibial component, proximal tibia, femoral component, and distal femur from 3D Slicer. Segmentations were taken from the seated WBCT scan. The coordinate system used for the current study is shown, with arrows indicating positive directions.

**Figure 4:**
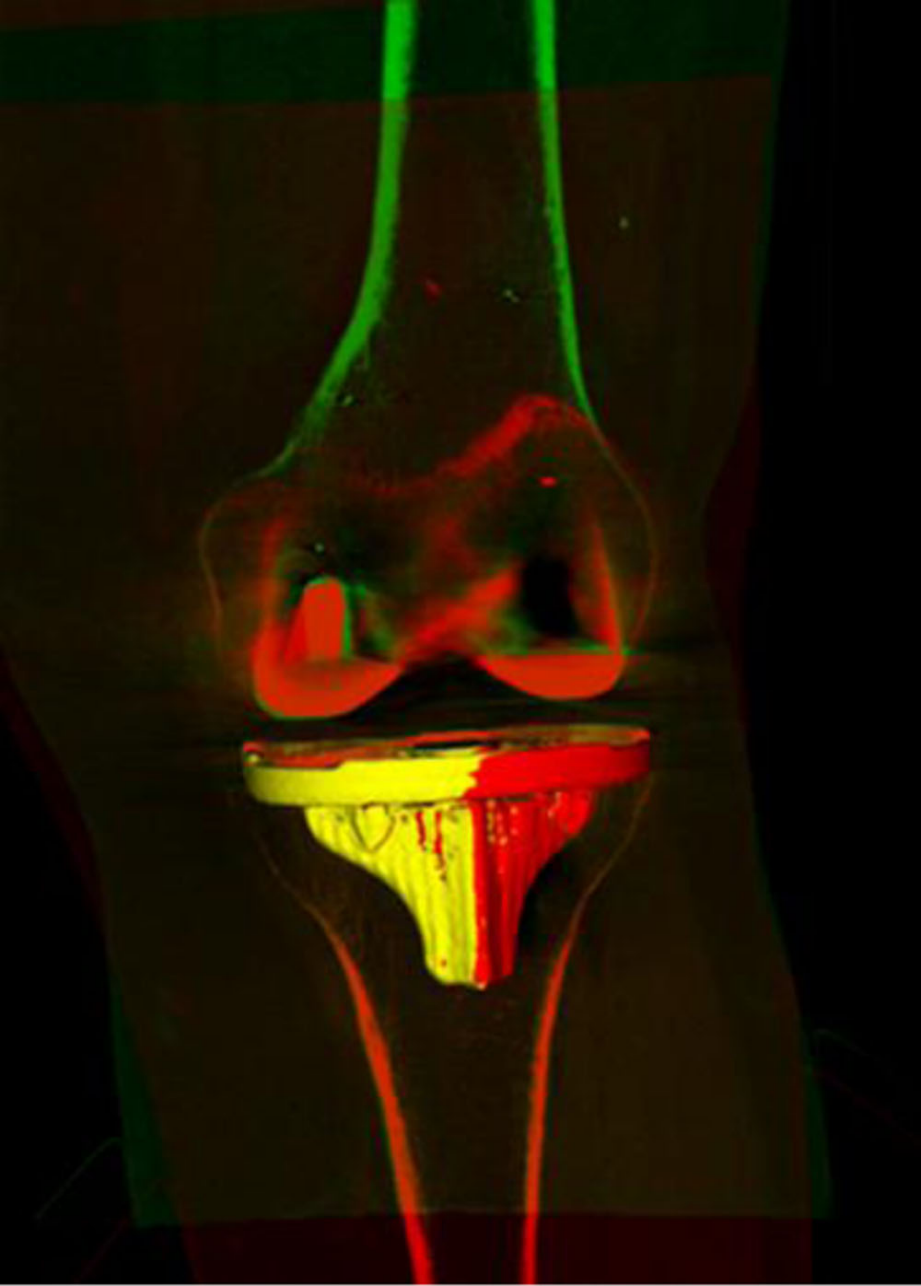
The change in tibial component position calculated by WBCT‐RSA. The red tibial component shows the initial position, and yellow tibial component is the migrated component showing the change in position.

For WBCT‐RSA, an open‐source segmentation software (Slicer.org, v5.2.2) was used to manually segment the tibial and femoral components, proximal tibia, and distal femur from the seated WBCT scan volumes. The scissors tool was used for segmentation by defining a region of interest (ROI) around the component or bone of interest which was then segmented by using a threshold based on component intensity in the ROI. Logical operators were used to ensure there was no overlap between implant component and its bone. The seated WBCT (reference scan), and standing WBCT (migrating scan), were loaded into Volumetric Matching Micromotion Analysis (V3MA), a novel CT‐RSA software (RSAcore, Leiden, The Netherlands). Three points were manually placed in each WBCT scan at identical landmarks in the axial view using V3MA. The segmentations (Figure 3) were loaded into the software, with the tibia or femur being the reference object, and the tibial or femoral component being the migrating object. V3MA was used to match the reference and follow‐up images in three steps. First, an alignment superimposed the points previously placed in each scan to superimpose the reference (seated) and migrating (standing) images as an initial match. Second, a reference match superimposed the reference objects (bone) in each scan. Third, a migrating match superimposed the migrating objects in each scan (implant component). V3MA uses image registration using the segmentations as a mask for matching. Changes in implant position were calculated by V3MA as translations, rotations, and MTPM between double examinations or the first seated exam and standing exam (Figure 4). Precision was reported as 1.96 times the standard deviation of the measurements between seated double examinations. Measurements were adjusted for a right‐sided implant. WBCT uses a Digital Imaging and Communication in Medicine (DICOM) coordinate system, which is different than RSA. Before analysis, all V3MA measurements were converted to the RSA coordinate system as previously described.

Statistical analyses were completed with Prism v9.5.1 (GraphPad Software, La Jolla, CA). Paired *t*‐tests or Wilcoxon tests were used to compare data depending on its normality. Comparisons were made between inducible displacement measurements from WBCT‐RSA and MBRSA in each axis of translation and rotation (X, Y, Z), and for total translation, total rotation, and MTPM. Inducible displacement measurements from WBCT‐RSA or MBRSA were compared to their double examination measurements in each axis to assess if results were measurement error or signal in the respective axis. Precision between WBCT‐RSA and MBRSA was compared using Levene's test to assess variances between groups followed by a Welch's *t*‐test if the variances were significantly different. Statistical significance was set at *p* < 0.05.

## 3 RESULTS

**Figure 5:**
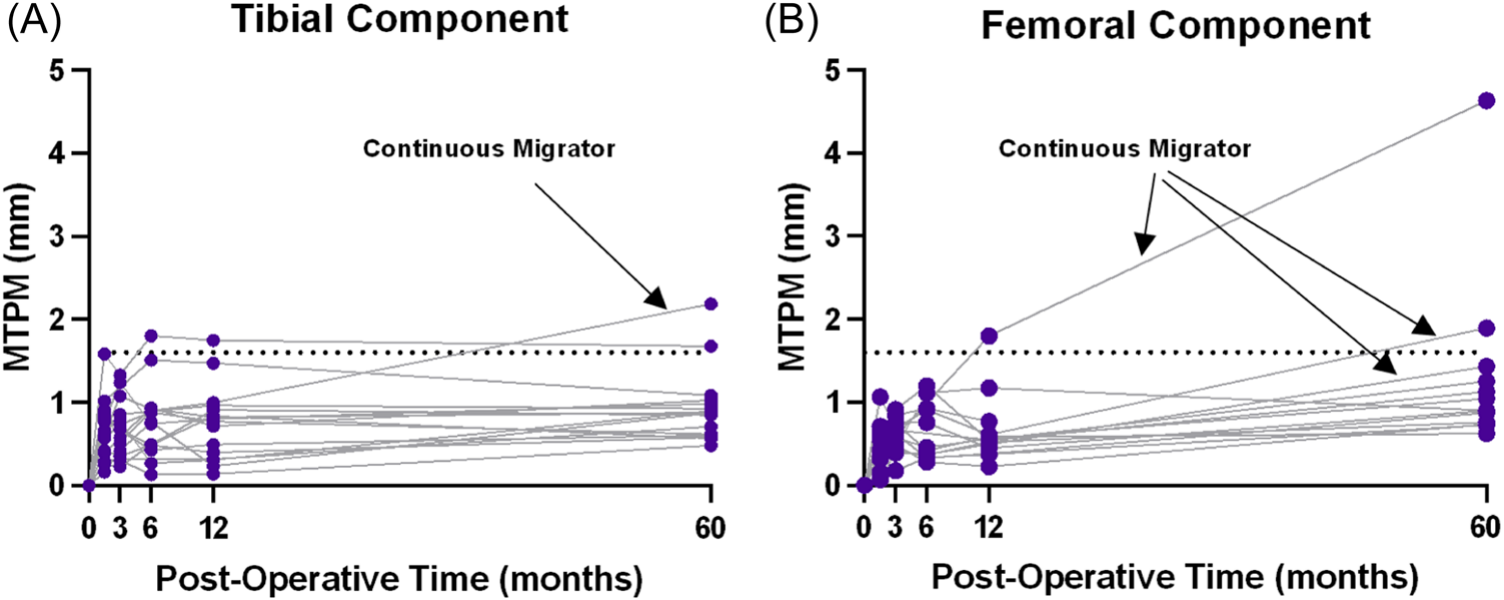
Longitudinal migration from 2 weeks to 5 years postoperation of the tibial (A) and femoral (B) components. Continuous migrators were determined based on a threshold of >0.20 mm migration per year from 1 to 5 years postoperation. Each line represents one participant.

The average yearly migration rate (MTPM) of the tibial component from 1 to 5 years postoperation was 0.08 ± 0.07 mm/year (Figure 5A). One tibial component had continuous migration of 0.30 mm/year, and inducible displacement at 5 years postoperation of 0.13 mm MTPM measured with WBCT‐RSA. The average yearly migration rate (MTPM) of the femoral component from 1 to 5 years postoperation was 0.18 ± 0.18 mm/year (Figure 5B). Three participants had yearly migration rates of the femoral component of 0.23 mm/year, 0.70 mm/year, and 0.32 mm/year, and inducible displacements of 0.79 mm, 0.97 mm, and 0.14 mm, respectively, at 5 years postoperation measured with WBCT‐RSA.

**Figure 6:**
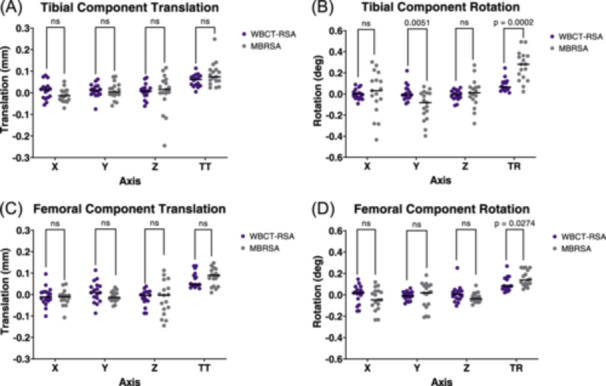
Translation and rotation measurements between double examinations for WBCT‐RSA (seated) and MBRSA (supine). Each point represents one participant. (A) Tibial component translation in each axis as well as total translation (TT). (B) Tibial component rotation in each axis as well as total rotation (TR). (C) Femoral component translation in each axis and TT. (D) Femoral component rotation in each axis and TR.

For the tibial component, precision of total translations with WBCT‐RSA and MBRSA were 0.056 and 0.108 mm (*p *> 0.05), respectively (Figure 6A). Precision of total rotations with WBCT‐RSA and MBRSA were 0.118° and 0.269° (*p *= 0.0002), respectively (Figure 6B). Precision of MTPM was 0.141 mm for WBCT‐RSA and 0.168 mm for MBRSA (Figure 8A, *p* = 0.005).

**Figure 7:**
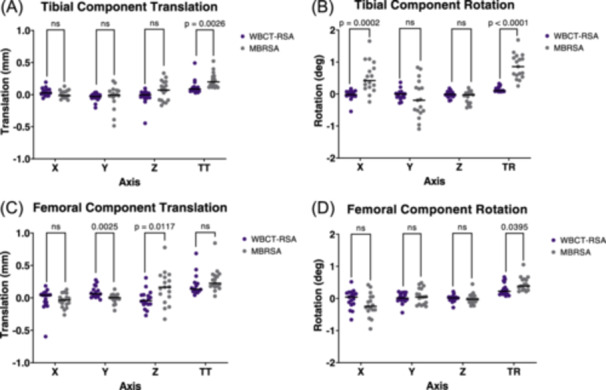
Inducible displacement translation and rotation measurements between WBCT‐RSA (seated to standing) and MBRSA (supine to standing). Each point represents one participant. (A) Tibial component translation in each axis and TT. (B) Tibial component rotation in each axis and TR. (C) Femoral component translation in each axis and TT. (D) Femoral component rotation in each axis and TR.

**Figure 8:**
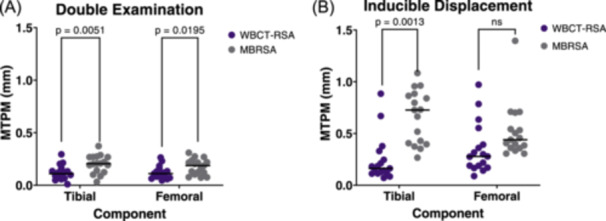
MTPM (mm) for double examinations (A) or inducible displacement examinations (B) between WBCT‐RSA and MBRSA. Each point represents one participant.

WBCT‐RSA and MBRSA measured significantly different inducible displacement measurements of the tibial component for total translation (Figure 7A, *p* = 0.003), anterior tilting (X axis rotation) (Figure 7B, *p* = 0.0002), total rotation (Figure 7B, *p* < 0.0001), and MTPM (Figure 8B, *p* = 0.001).

With WBCT‐RSA, inducible displacement measurements of the tibial component were significantly different from the double examinations for subsidence (Y axis translation) (*p *= 0.004), total translation (*p *= 0.004), total rotation (*p *= 0.034), and MTPM (*p *= 0.013) (Table 1). With MBRSA, significant differences were found for total translation (*p *= 0.0004), anterior tilting (X axis rotation) (*p *= 0.0003), varus‐valgus (Z axis) rotation (*p *= 0.0180), total rotation (*p *< 0.0001), and MTPM (*p *< 0.0001) (Table 1).

**Table 1:** Mean and 95% CI of double examinations and inducible displacement (ID) exams with both MBRSA and WBCT‐RSA. Measurements are reported for the tibial component in each axis of translation and rotation, and for total translation (TT) and total rotation (TR).

		MBRSA								
		Translation (mm)				Rotation (°)				
Exam	Value	X	Y	Z	TT	X	Y	Z	TR	MTPM
Double exam	Mean	−0.013	0.009	0.002	0.085	0.010	−0.108	0.006	0.264	0.203
	CI	(−0.029, 0.003)	(−0.012, 0.029)	(−0.044, 0.047)	(0.056, 0.113)	(−0.096, 0.117)	(−0.179, −0.036)	(−0.063, 0.076)	(0.193, 0.334)	(0.159, 0.247)
ID	Mean	0.004	−0.037	0.064	0.223	0.530	−0.152	−0.099	0.866	0.662
	CI	(−0.027, 0.035)	(−0.131, 0.056)	(−0.016, 0.144)	(0.163, 0.283)	(0.282, 0.777)	(−0.454, 0.150)	(−0.196, −0.002)	(0.669, 1.064)	(0.530, 0.794)
*p*‐value		>0.05	>0.05	>0.05	0.0004	0.0003	>0.05	0.0180	<0.0001	<0.0001

		WBCT‐RSA								
		Translation (mm)				Rotation (°)				
Exam	Value	X	Y	Z	TT	X	Y	Z	TR	MTPM
Double exam	Mean	0.010	0.011	0.007	0.057	0.000	0.011	–0.012	0.085	0.120
	CI	(−0.011, 0.032)	(−0.007, 0.028)	(−0.012, 0.026)	(0.042, 0.072)	(−0.027, 0.027)	(−0.030, 0.053)	(−0.038, 0.015)	(0.053, 0.117)	(0.081, 0.158)
ID	Mean	0.043	−0.038	−0.034	0.115	−0.021	−0.017	−0.016	0.151	0.244
	CI	(0.011, 0.074)	(−0.070, −0.006)	(−0.095, 0.026)	(0.059, 0.172)	(−0.103, 0.060	(−0.098, 0.064)	(−0.067, 0.035)	(0.107, 0.196)	(0.131, 0.357)
*p*‐value		>0.05	0.0042	>0.05	0.0042	>0.05	>0.05	>0.05	0.0335	0.0131

For the femoral component, precision of total translations with WBCT‐RSA and MBRSA were 0.071 and 0.080 mm (*p *> 0.05), respectively (Figure 6C). Precision of total rotations with WBCT‐RSA and MBRSA were 0.128° and 0.144° (*p *= 0.027), respectively (Figure 6D). Precision of MTPM was 0.117 mm for WBCT‐RSA and 0.146 mm for MBRSA (Figure 8A, *p* = 0.020).

WBCT‐RSA and MBRSA measured significantly different inducible displacement measurements of the femoral component for superior‐inferior (Y axis) translation (Figure 7C, *p* = 0.003), anterior‐posterior (Z axis) translation (Figure 7C, *p*= 0.012), and total rotation (Figure 7D, *p* = 0.040).

With WBCT‐RSA, inducible displacement measurements of the femoral component were significantly different from the double examinations for subsidence (Y axis translation) (*p *= 0.009), total translation (*p *< 0.0001), total rotation (*p *= 0.0003), and MTPM (*p *< 0.0001) (Table 2). With MBRSA, significant differences were found for anterior‐posterior (Z axis) translation (*p *= 0.010), total translation (*p *= 0.0002), total rotation (*p *< 0.0001), and MTPM (*p *< 0.0001) (Table 2).

**Table 2:** Mean and 95% CI of double examinations and inducible displacement (ID) exams with both MBRSA and WBCT‐RSA. Measurements are reported for the femoral component in each axis of translation and rotation, and for TT and TR.

		MBRSA								
		Translation (mm)				Rotation (°)				
Exam	Value	X	Y	Z	TT	X	Y	Z	TR	MTPM
Double exam	Mean	−0.014	−0.011	−0.016	0.081	−0.042	−0.007	−0.025	0.154	0.177
	CI	(−0.033, 0.005)	(−0.023, 0.002)	(−0.057, 0.024)	(0.060, 0.102)	(−0.095, 0.012)	(−0.070, 0.056)	(−0.048, ‐0.002)	(0.116, 0.191)	(0.138, 0.215)
ID	Mean	−0.044	−0.008	0.155	0.274	−0.206	0.067	−0.002	0.454	0.520
	CI	(−0.099, 0.011)	(−0.047, 0.031)	(0.022, 0.288)	(0.182, 0.366)	(−0.390, ‐0.022)	(−0.061, 0.195)	(−0.085, 0.081)	(0.342, 0.566)	(0.385, 0.656)
*p*‐value		>0.05	>0.05	0.0103	0.0002	>0.05	>0.05	>0.05	<0.0001	<0.0001

		WBCT‐RSA								
		Translation (mm)				Rotation (°)				
Exam	Value	X	Y	Z	TT	X	Y	Z	TR	MTPM
Double exam	Mean	−0.008	0.012	−0.014	0.068	0.002	−0.011	0.006	0.102	0.122
	CI	(−0.031, 0.014)	(−0.013, 0.037)	(−0.033, 0.005)	(0.049, 0.086)	(−0.042, 0.047)	(−0.031, 0.010)	(−0.034, 0.046)	(0.068, 0.136)	(0.091, 0.152)
ID	Mean	−0.022	0.089	−0.028	0.197	−0.019	0.011	−0.004	0.282	0.355
	CI	(−0.111, 0.067)	(0.043, 0.135)	(−0.100, 0.044)	(0.115, 0.280)	(−0.165, 0.127)	(−0.072, 0.095)	(−0.061, 0.052)	(0.185, 0.379)	(0.228, 0.481)
*p*‐value		>0.05	0.0087	>0.05	<0.0001	>0.05	>0.05	>0.05	0.0003	<0.0001

## 4 DISCUSSION

Aseptic loosening remains one of the top causes of late revision surgery.^2^, ^3^ RSA is currently used to assess implant fixation in research, however its limitations prompted the use of widely available CT scanners to perform RSA‐like measurements.^7^, ^10^, ^11^ This study aimed to determine inducible displacement measurements of TKA components with a novel WBCT‐RSA approach compared to conventional RSA. We found comparable precision between WBCT‐RSA and MBRSA for all axes of translation and rotation of both the tibial and femoral component, except for tibial component Y axis rotation. Significant differences in precision between the two imaging techniques were found for total rotations and MTPM of both components. Where there were differences, WBCT‐RSA had better precision than MBRSA. All inducible displacement measurements in this study were below previously established thresholds that indicate a probability that the implant is loose, suggesting these results are for a cohort of stable implants.^19^, ^21^, ^31^

The mean yearly migration rate from 1 to 5 years postoperation for the tibial components was less than 0.2 mm/year, which is not considered to be clinically relevant.^32^ All but one tibial component was below this value. MTPM < 0.2 mm per year after 2 years postoperation indicates stable components and is predictive of long‐term tibial component survival.^8^ The single tibial component above the threshold had only 0.30 mm/year in longitudinal MTPM and inducible displacement MTPM at 5 years postoperation of 0.13 mm measured with WBCT‐RSA, suggesting this component is not grossly loose. This may also suggest that although an implant migrates over time, inducible displacement exams may not cause implant movement. Inducible displacement values up to 1.7 mm for MTPM have been reported for stable tibial components, and all measurements in the current study were well below this.^19^, ^21^, ^31^ There were, however, one tibial, and three femoral components above previously established thresholds ranges for stable longitudinal migration. If WBCT‐RSA is more precise than MBRSA and is measuring stable inducible displacement values, this may suggest that thresholds for longitudinal migration may be redefined in the future.

There are currently no thresholds to predict the long‐term survival of femoral components. Three components in this study appear to be continuously migrating, and two of them had inducible displacement values of 0.79 mm and 0.97 mm with WBCT‐RSA, almost twice as high as the group mean. The third component had an inducible displacement of 0.14 mm, suggesting it may not be grossly loose. These results show that femoral inducible displacement was much greater than the precision measurements, suggesting there was movement present. However, the tibial component is more frequently studied and without thresholds for femoral longitudinal migration or inducible displacement, it is difficult to make conclusions about the fixation of these components.^33^

Several studies have compared CT‐RSA and MBRSA. A phantom study for tibial components found better precision with CT‐RSA, with a precision of 0.08 mm for total translation with CT‐RSA, and 0.58 mm for MTPM with MBRSA.^26^ Another study found precision of acetabular cups to be 0.10–0.16 mm for translation and 0.21–0.31° for rotation with CT‐RSA, and 0.09–0.26 mm for translation and 0.43–1.69° for rotation with marker‐based RSA.^11^ We found precision values lower (meaning better) than these previous studies, and all values were below 0.50 mm supporting the use of this novel WBCT‐RSA approach due to its ability to detect clinically important migration.^26^ Specifically, a study by de Laat et al. found that both accuracy and precision of V3MA, the CT‐RSA software used for the current study, were comparable to RSA for tibial component migration.^15^ Our results support this study and also include precision values for femoral component migration. The current study and work by de Laat show promising results for V3MA to be used as a clinical research tool.

Significant differences were seen between WBCT‐RSA and MBRSA for total translation, total rotation, and MTPM. Due to the variability in the point chosen for MTPM, and error in individual planes being multiplied for total translation and rotation results, it is likely more valuable to compare individual axes. Comparable precision was found in every axis of translation and rotation for both components, except for tibial component Y axis rotation. For inducible displacement measurements, significant differences were seen in the Y and Z axes for translations, and X axis for rotations. When comparing the inducible displacement examinations to the double examinations, MBRSA measured significant signal for anterior‐posterior (Z axis) translation of the femoral component, and anterior tilt (X axis rotation) and Z axis rotation of the tibial component. For WBCT‐RSA, significant signal was found for subsidence (Y axis translation), with predominantly superior translation of the femoral component and inferior translation of the tibial component, as would be expected under compressive axial loading from standing. All measurements were small (<1.50 mm), however induced anterior tilt measured with MBRSA was much higher than the other axes with 0.530° of rotation. This would also cause total rotation and MTPM to increase and may suggest the influence of the loading protocol. A study by Buckley et al., found that the force through the heels when lying supine on a hard surface can create a force on the knee equivalent to that when standing, and hanging feet off the edge of the surface completely removes this force on the knee.^34^ Our study used the same supine position for the RSA examinations and could suggest that the additional force on the knee when lying supine may influence the starting position of the implant and alter the observed translations, in comparison to WBCT‐RSA where the unloaded position was seated with the leg elevated in approximate extension.

It should be noted that MBRSA measured significantly higher total rotations and anterior tilt (X‐axis rotation) of the tibial component than WBCT‐RSA, suggesting possible error with rotation measurements. For accurate results, MBRSA relies heavily on the registration process of the tibial or femoral components.^35^ If the components are oriented to where their coordinate system aligns with the laboratory coordinate system, unique features of the model may be hidden and MBRSA will have difficulty with registration, causing increased error. The tibial component is symmetrical, making it even more important to orient the baseplate to where unique features are visible.^35^, ^36^ WBCT‐RSA measured comparable precision to MBRSA in all axes of translation and rotation, except for Y axis rotation, where WBCT‐RSA had better precision. This may suggest the benefit of WBCT‐RSA to more accurately register the implant components, especially with symmetrical implants.

This study is not without limitations. As RSA studies typically recruit a small number of participants, there are only 17 participants in the present study, although this surpasses the 15 required for precision measurements.^26^ Four subjects were excluded from the longitudinal migration analysis with MBRSA due to bead alignment. However, all had inducible displacement measurements within safe threshold ranges suggesting the components are stable and may also further support the use of WBCT‐RSA to eliminate the reliance on bone markers. The loading protocol was not identical with each technique, however it was similar, and the comparable precision of WBCT‐RSA and MBRSA and inducible displacement measurements showing stable components is encouraging for future studies using WBCT‐RSA to assess fixation. There were several femoral components that appear to be continuously migrating but without longitudinal migration thresholds for the femoral component, it is difficult to form conclusions. It would be beneficial to examine subjects with components that are known to either be well‐fixed or loose at the time the images are acquired to determine clinical thresholds, and the precision of WBCT‐RSA found in this study support its future use for those measurements. WBCT is a more expensive technology, therefore translation to clinical use may be a long process. With the increasing availability of WBCT, its comparable precision to RSA, and its ability to measure other parameters with a single radiation exposure, its current use in research is valuable.

This study is the first to clinically compare WBCT‐RSA to MBRSA to assess TKA components. WBCT‐RSA and MBRSA inducible displacement measurements were below previous thresholds indicating the implant is likely loose, and along with the longitudinal migration findings, suggest that the range of inducible displacements measured here represent that of well‐fixed components. This study prompts future studies using WBCT‐RSA to assess inducible displacement of suspected loose components. The precision of WBCT‐RSA shows its ability to be used in the future to assess the instantaneous fixation of implant components.

## AUTHOR CONTRIBUTIONS

Matthew G. Teeter designed the study. Rebecca A. Hext was responsible for data measurement, statistical analysis, and interpretation of results. Bart L. Kaptein developed and provided the software for analyzing weight‐bearing computed tomography images. Rebecca A. Hext drafted the manuscript and all authors contributed to revising the manuscript. All authors have read and approved of the submitted manuscript.
